# Fine-tuning immunity: ubiquitin-dependent regulation of interleukin-17A expression by Th17 cells

**DOI:** 10.3389/fimmu.2025.1614409

**Published:** 2025-08-01

**Authors:** Desh Raj, Amit K. Verma, Risha Mathur, Jenna Woody, Mahesh Kathania, K. Venuprasad

**Affiliations:** ^1^ Department of Internal Medicine, UT Southwestern Medical Center, Dallas, TX, United States; ^2^ Department of Immunology, UT Southwestern Medical Center, Dallas, TX, United States; ^3^ Harold C. Simmons Comprehensive Cancer Center, UT Southwestern Medical Center, Dallas, TX, United States

**Keywords:** IL-17A, ubiquitination, deubiquitination, RORgt, E3 ligases

## Abstract

Interleukin-17A (IL-17A) is a pro-inflammatory cytokine that plays a pivotal role in immune responses, particularly in the pathogenesis of various autoimmune diseases and infections. Recent advances have highlighted the significance of post-translational modifications, particularly ubiquitination, in regulating IL-17A expression and IL-17A receptor signaling pathways. Here, we summarize the intricate relationship between IL-17A and ubiquitination, exploring how ubiquitin-mediated processes influence IL-17A production, receptor signaling, and downstream effector functions. We provide insights into the potential therapeutic implications of targeting IL-17A and its ubiquitination pathways in inflammatory diseases and autoimmune disorders. A clear understanding of this relationship could pave the way for novel strategies in immune modulation, potentially enhancing management and treatment efficacy in various human diseases.

## Introduction

Interleukin 17A (IL-17A) is critical in the host immune response against bacterial and fungal infections, especially at the mucosal surface (1, 2). However, dysregulated IL-17A expression is strongly linked to several human diseases, such as multiple sclerosis (MS), psoriasis, systemic lupus erythematosus (SLE), rheumatoid arthritis (RA), and asthma (2). Its biological effects are mediated by activating various signaling pathways that regulate the transcription of target genes involved in inflammation, tissue remodeling, and the recruitment of immune cells (2).

Ubiquitination, a post-translational modification whereby ubiquitin moieties are covalently attached to target proteins, regulates numerous cellular processes, including protein degradation, signal transduction, and cellular localization (3). It involves a multi-enzymatic biochemical reaction in which the Ub-activating (E1) enzyme activates Ub, which is then transferred to the Ub-conjugating (E2) enzyme. The Ub-ligating (E3) enzymes facilitate the formation of the isopeptide bond between the Ub (C-terminus) and specific substrate lysine residues (3). The human genome is estimated to encode 2 E1s, nearly 50 E2s, and over 600 E3s. Each E3 recognizes a set of substrates that share one or more ubiquitination signals, and an individual E3 cooperates with one or a few E2s. The E3 ligases are divided into two different groups based on their functional domains: the homology to the E6-associated protein carboxyl terminus (HECT) type E3s and the fascinating new gene (RING) type E3s (3). The ubiquitin molecules in a polyubiquitin chain are generally linked through the K48 or K63-linked polyubiquitin chains; however, other lysine residues in a ubiquitin molecule have been shown to participate in linkage (3). Interestingly, the different types of polyubiquitin chains have different effects on the substrate. The function of E3 ubiquitin ligases is reversed by the action of deubiquitinating enzymes (DUBs) (4). They specifically cleave the isopeptide bonds between ubiquitin and the Lys residue within the ubiquitinated substrate (4). The dynamic interplay between ubiquitination and the signaling pathways that lead to IL-17 expression is an area of growing interest, offering insights into the fine-tuning of immune responses. Ubiquitin ligases and deubiquitinases modulate the expression of IL-17A and the stability and activity of IL-17A signaling components, thereby influencing the intensity and duration of IL-17A-mediated cellular responses.

Here, we summarize the current knowledge regarding the relationship between IL-17A and ubiquitination, highlighting how this axis regulates immune responses and the implications for therapeutic interventions in inflammatory diseases.

## IL-17 and inflammation

The IL-17 family of cytokines consists of six members (IL-17A to IL-17F) which binds to IL-17 receptors (IL-17RA to IL-17RE) and implement their physiological functions (5). Among the IL-17 family members, IL-17A is the most studied and highly significant cytokine. The human IL-17A is synthesized as a 155-amino-acid precursor which is then posttranscriptional modified by cleave of 23-amino-acid signal peptide at the N-terminus which is followed by dimerization via disulfide bonds to generate mature homodimer of 35 kDa (6). Among the members of the IL-17 family, IL-17F is most similar to IL-17A, with 55% sequence homology (7). IL-17F forms homodimers or heterodimers with IL-17A and binds to IL-17 receptors for signal transduction.

IL-17A is predominantly expressed by CD4^+^ T helper cells (Th17); however, natural killer T cells, CD8^+^ T cells, γδ T cells, innate lymphoid cells (ILCs), dendritic cells, macrophages, and other cells also produce this cytokine (8). The differentiation of Th17 cells depends on the presence of proinflammatory Interleukin-6 (IL-6), Transforming growth factor-beta (TGF-β), and Interleukin 1β (IL-1β) (8, 9). Th17 differentiation requires the activation of the transcription factors signal transducer and activator of transcription 3 (STAT3) and retinoic acid-related orphan receptor gamma t (RORγt) (10). After the initial differentiation, Th17 expresses the IL-23 receptor (IL-23R) and requires IL-23 for their proliferation and survival. Although T-cell receptor (TCR) activation is necessary for CD4^+^ and CD8^+^ T-cell IL-17A synthesis, innate immune cells primarily produce IL-17A in the presence of IL-6 and IL-23 (11).

IL-17A is a potent proinflammatory cytokine that induces neutrophil and monocyte recruitment to the site of inflammation by inducing the expression of chemokine (C-X-C motif) ligand 1 (CXCL1), CXCL2, and CCL20 (8). IL-17A also promotes neutrophil differentiation via the production of granulocyte colony-stimulating factors (G-CSF) and monocyte chemoattractant protein-1 (MCP-1) by non-hematopoietic target cells (11). *Il17a* deficiencies in mice result in defective neutrophils, leading to increased susceptibility to extracellular pathogens, including the bacteria *Klebsiella pneumoniae*, *Candida albicans*, and *Toxoplasma gondii* (12). In addition, IL-17A regulates the expression of molecules with antimicrobial activity, such as β-defensins, calgranulins, and mucins. Defensins act as natural antibiotics in the lungs, skin, and gut. Another IL-17A target gene is Chemokine (C-C motif) ligand 20 (CCL20), a chemokine that recruits dendritic cells (DCs) and T cells, thereby providing a positive feedback loop for IL-17A amplification by recruiting Th17 cells to inflamed sites (12). However, IL-17A is not always protective against infections. In schistosomiasis, IL-17A stimulates a pathogenic inflammatory response that can be alleviated with antibodies to IL-17A (13). Elevated IL-17A levels are also associated with severe periodontal disease. Importantly, elevated IL-17A is strongly linked to autoimmune pathology. Increased IL-17A levels were found in RA, SLE, and psoriasis patients (11). Consistent results suggest a pathogenic role for IL-17A in various mouse models of autoimmune disease. Similarly, dysregulated IL-17A-mediated inflammation is linked to graft vs host disease and some cancers (14, 15).

## Intracellular events of IL-17A expression

The differentiation of Th17 cells requires coordinated activation of T cells in the presence of TGF-β, IL-6, IL-1β, and IL-23 (8). TCR stimulation-induced phosphatidylinositol 3-kinase (PI3K), Nuclear factor kappa-light-chain-enhancer of activated B cells (NF-κB), Nuclear Factor of Activated T cells (NFAT), and Mitogen-activated protein (MAP) kinase pathways are involved in IL-17A production (10). RAR-related orphan receptor gamma t (RORγt), a member of the nuclear receptor family of proteins, is a key transcriptional factor for IL-17A expression (16). It has been demonstrated that cholesterol derivatives, including desmosterol and oxysterols, serve as natural ligands and activate RORγt (17, 18). Whereas 3-oxoLC, a bile acid synthesized from cholesterol, acts as an inhibitory ligand of RORγt (19). Further, Raftlin1, a lipid raft protein, was shown to recruit specific phospholipids to RORγt and promote the transcriptional activity of RORγt and IL-17A expression (20). RORγt binds to RORE sequences within the CNS2 of the *Il17a* gene and mediates *Il17a* transcription by controlling the chromatin remodeling (10). In addition to RORγt, p300 and JmjC domain-containing protein (JMJD)3 also bind to CNS2 and mediate permissive histone acetylation and remove repressive histone marker H3K27me3 (21). CNS2 interacts with the Il17a promoter to induce Il17a transcription (10, 22). Runt-related transcription factor (RUNX)1 also binds to the CNS2 region of *Il17a* promoter (23). RUNX1 binds to RORgt to enhance expression of *Il17*a (10, 24).

STAT3 (another transcription factor), activated by IL-6, is involved in IL-17A expression by binding to the the Il17a promoter (25). Additionally, JunB was found to colocalize with interferon regulatory factor (IRF)4, which is involved in IL17A expression (26, 27). IRF4 binds to the regulatory elements of the Il17a promoter, which are co-bound by BATF, an AP-1 factor (10, 27). KLF4, a Kruppel-like factor, is involved in IL-17A expression by directedly binding to the Il17a promoter independently of RORgt (28). A metabolic sensor, Hypoxia-inducible factor (HIF)-1α, associates with RORgt, and binds to hypoxia response element located in the proximal region of the *Rorc* promoter (29). This suggests a complex network of transcriptional regulators is involved in generating Th17 cells.

## Ubiquitination in IL-17A expression

**Figure 1:**
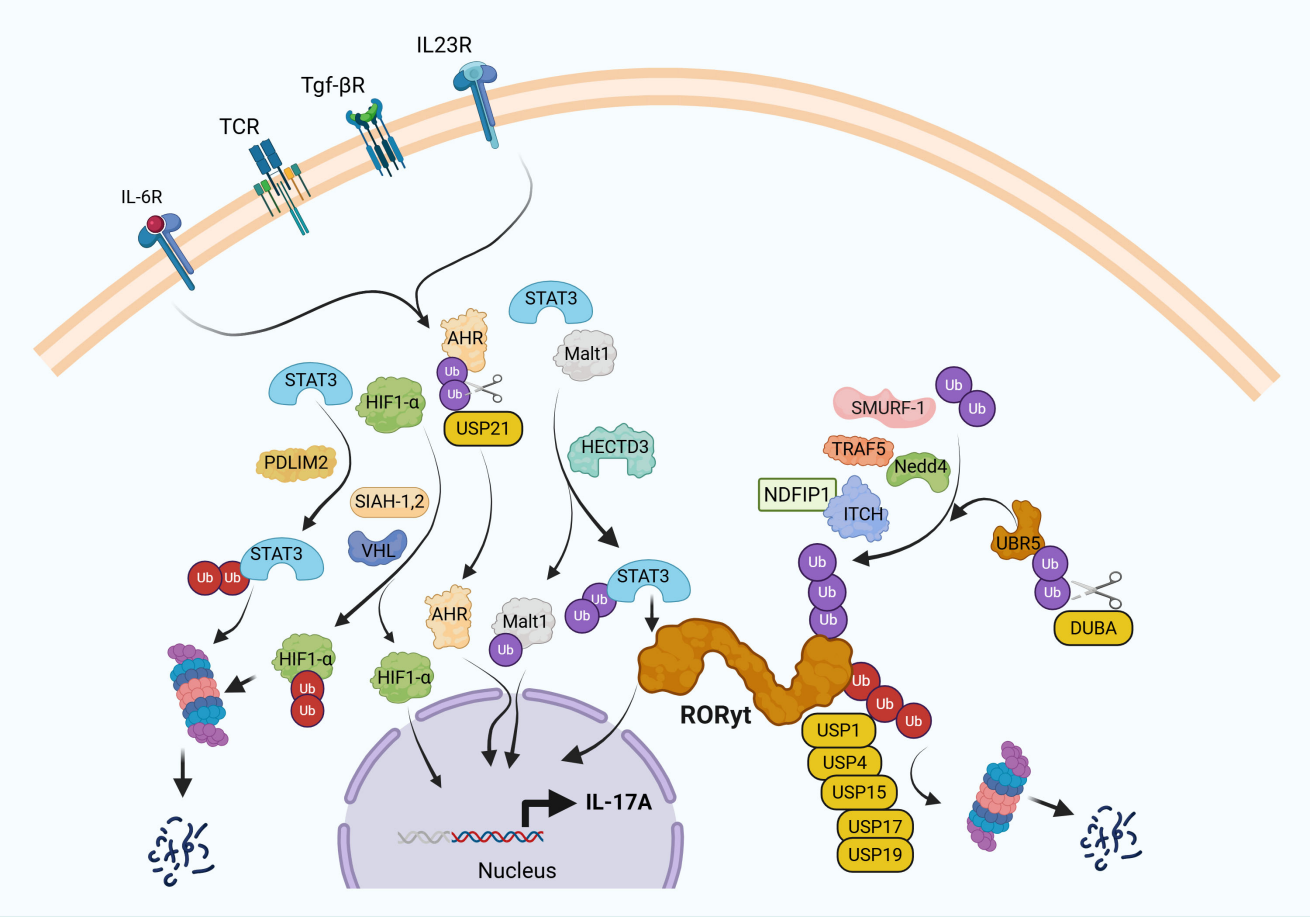
Ubiquitin pathway regulates IL-17A expression in Th17 cells. T cell receptor (TCR) ligation in the presence of IL-6, TGF-β, and IL-23 induces differentiation of naïve CD4^+^ T cells into Th17 cells through activation of key transcription factors, including RORγt, aryl hydrocarbon receptor (AHR), MALT1, and HIF-1α, which collectively drive IL-17A expression. Ubiquitin-mediated post-translational modifications tightly control this process. E3 ubiquitin ligases such as Itch and SMURF1 target RORγt for ubiquitination, thereby limiting chronic IL-17A production. In contrast, TRAF5 and NEDD4 promote IL-17A expression, supporting Th17 polarization. HECTD3 enhances Th17 pathogenicity by ubiquitinating STAT3 and MALT1, while SLIM/PDLIM2 facilitates proteasomal degradation of STAT3. SIAH1/2 stabilizes HIF-1α to promote Th17 differentiation, whereas the E3 ligase VHL paradoxically also contributes to Th17 development by ubiquitinating HIF-1α for degradation, reflecting the context-dependent roles of these factors. Deubiquitinases (DUBs) further fine-tune this regulatory network. DUBA interacts with UBR5 to modulate RORγt ubiquitination, acting as a negative regulator of IL-17A expression. Additional DUBs, including USP1, USP4, USP15, and USP17, contribute to the precise control of RORγt activity. USP21 stabilizes AHR, thereby indirectly enhancing Th17 differentiation. These findings underscore the complexity and specificity of ubiquitin-mediated regulation in IL-17A-driven immune responses, highlighting potential targets for therapeutic intervention in autoimmune diseases.

Ubiquitin(Ub) conjugation was initially thought to be involved in proteasomal degradation of misfolded proteins (30). However, increasing evidence shows a broader implication in multiple subcellular processes, including the localization of proteins withing the cytoplasm, nuclear translocation, protein-protein interactions, cell membrane receptor turnover, and gene expression. Predictably, IL-17A expression is also regulated by ubiquitin conjugation (**Figure 1**).

### E3 Ligases and IL-17A

The PDZ-LIM domain protein PDLIM2, a nuclear ubiquitin E3 ligase, has been shown to inhibit Th17 cells by targeting STAT3 for polyubiquitination and proteasomal degradation (31, 32). Deficiency in PDLIM2 resulted in the accumulation of STAT3 in the nucleus, enhanced Th17 cell differentiation, and exacerbated IL-17A-mediated granuloma formation (32). whereas the E3 ubiquitin ligase HECTD3 promoted Th17 cells via non degradative K27-linked and K29-linked polyubiquitin chains on STAT3 and Malt1 (33). *Hectd3*-deficient mice exhibited reduced EAE severity and defective Th17 cell differentiation (33).

E3 ubiquitin protein ligase Itch regulates IL-17A production by ubiquitination of RORgt in Th17 cells (34). Itch recognizes the PPLY region on RORgt through its WW domain resulting in proteasomal degradation leading to inhibition of IL-17A expression. Itch deficiency resulted in spontaneous dermatitis and colitis, which was associated with elevated IL-17A expression (34). A defect in ITCH-mediated RORγt degradation was demonstrated in colorectal cancer (CRC) patients, where Colon rectal neoplasia differentially expressed(CRNDE-h ) protein was shown to associate with the conserved PPLY region within RORgt in Th17 cells infiltrated to tumors (35). CRNDE-h binding of to RORgt prevented ubiquitination of RORgt by blocking its binding to ITCH (35). Not surprisingly, a the percentage of Th17 cells among tumor-infiltrating lymphocytes (TILs) from CRC patients was positive correlated with CRNDE-h expression (35). This further supported the observed aggressive colon cancer growth in Itch−/− mice. This data further highlighted the involvement of ITCH in Th17-mediated tumor-promoting inflammation. Another report showed that Nedd4 targets RORγt for K27-linked ubiquitination, which promotes IL-17A, and *Nedd4* deficiency resulted in attenuated IL-17A production and EAE (36). Further, HECTD3 family interacting protein 1 (Ndfip1), a co-activator of the E3 ubiquitin ligase Itch, attenuates the frequency and Th17 cells pathogenicity (37, 38). NDFIP1 binds to Itch and promotes its ligase activity in murine CD4^+^ T cells following TCR ligation via recruitment of ubiquitin-conjugating enzyme E2 (UBCH7) to Itch (39). Similar to *Itch* ^−/−^ Th17 cells, when adoptively transferred, Ndfip1 deficient Th17cells produced more IL-17A and induced severe colitis, indicating a pivotal role for the NDFIP1-ITCH pathway in the regulation of IL-17A-mediated inflammation (37, 38).

What triggers the Itch-mediated ubiquitination of RORγt? It was shown that p21-activated kinase 2 (Pak2), a serine (S)/threonine (T) kinase, was shown to recognize a conserved KRLS motif within RORγt and phosphorylates the S-316 within this motif (40). Pak2-mediated phosphorylation enhanced RORγt ubiquitination. The genetic deletion of Pak2 in Th17 cells reduces RORγt phosphorylation and increases IL-17A expression. Similarly, the reconstitution of RORγt-S316A mutant in *Rorc* ^−/−^ Th17 cells enhanced IL-17A expression due to reduced ubiquitination (40). In silico analysis of the modeled structure of RORγt showed that S316 makes an H-bond (3.6 Å) with a side-chain amino group of asparagine (N) 253 of the neighboring α-helix and stabilizes the ligand-binding domain (LBD) of RORγt, which reduces the accessibility of the PPLY motif. When S316 was substituted with phospho-mimetic aspartic acid residues (D) 316, the H-bond interaction between S316 and N253 was abolished, suggesting that phosphorylation provides increased accessibility of the PPLY motif of RORγt to ITCH (40). This suggested that a crosstalk between phosphorylation and ubiquitination plays a critical role in regulating the stability of RORγt and IL-17A expression. While Itch promoted degradation of RORγt, tumor necrosis factor receptor-associated factor 5 (TRAF5) interacts with and ubiquitinates RORγt via Lys-63-linked polyubiquitination (41). TRAF5 stabilizes the RORγt protein level depending on its RING finger domain. Depletion of TRAF5 in Th17 cells destabilizes RORγt protein and downregulates Th17-related genes, including *Il17a* (41).

Screening of a cDNA library to identify specific modulators for *Il17a* promoter activity led to the identification of the E3 ubiquitin ligases SIAH1 and SIAH2, as positive regulators of Il17a promoter activity in a T-cell line and promoted Th17 development *ex vivo* (42). This enhancement was a consequence of increased HIF-1α protein. Without HIF-1α, both ubiquitin ligases had little effect on Th17 cell differentiation (42). These results suggest that SIAH1 and SIAH2 play a pivotal role in promoting Th17 cell differentiation via the modulation of the stability of HIF-1α protein. Also, deletion of von Hippel-Lindau (VHL), an E3 ubiquitin ligase that targets HIF-1α, promoted Th17 differentiation (43). Mice deficient in VHL in their T cells were resistant to EAE. In the absence of VHL, Th17 cells had decreased activation of STAT3 and SMAD2 (43) (**Table 1**).

**Table 1:** List of E3 ligases and DUBs and their function in IL-17A expression.

E3 ligase	Function	Disease model	Ref.
ITCH, NDFIP1, and UBR5.	Promotes degradation of RORγt	IBD, EAE, RA.	(34, 36–38, 40, 44)
TRAF5 and SMURF1.	Stabilizes RORγt, leading to enhanced IL-17A expression	EAE	(41, 45)
PDLIM2	Attenuates Th17 cell differentiation by targeting STAT3 for degradation.	EAE	(31, 32)
HECTD3	Necessary for pathogenic Th17 cell generation by promoting polyubiquitination of STAT3 and Malt1.	EAE	(33)
NEDD4	This E3 ligase enhances RORγt activity during Th17 cell development by catalyzing K27-linked polyubiquitination.	EAE	(36)
Von Hippel-Lindau (VHL)	Promotes Th17 differentiation and regulates cellular metabolism in Th17 cells.	EAE	(43)
Deubiquitinating enzymes (DUBs)	Function	Disease model	Ref.
DUBA (Deubiquitinase A)	Stabilizes UBR5	Autoimmune diseases	(44)
USP-1	Enhances RORγt activity while promoting Foxp3 degradation.	Inflammatory diseases	(46)
USP-4	Maintains RORγt function in Th17 cells.	Inflammatory disease	(47)
USP-15	Interacts with RORγt removing ubiquitin from K446 and stimulates RORγt activity by enhancing SRC1.	Autoimmune diseases.	(48)
USP-17	Stabilizes RORγt protein expression by reducing polyubiquitination at Lys-360.	Systemic lupus erythematosus.	(49)
USP-19	Removes K63-linked ubiquitin from RORγt lysine 313, crucial for SRC3 coactivator recruitment.	EAE	(50)
USP-21	Deubiquitinates AhR at K432. Its deficiency enhances Th17 cell differentiation.	–	(51)

### DUBs and IL-17A

Ubiquitination is a highly dynamic and reversible process, and the removal of Ub chains bound to protein substrates is mediated by deubiquitinating enzymes (DUBs). DUBA is a deubiquitylating enzyme that negatively regulates IL-17A production in T cells (44). DUBA is associated with the UBR5 (a ubiquitin ligase), suppressing abundance of DUBA in naive T cells. Accumulated DUBA stabilized UBR5, which then ubiquitylated RORgt in response to TGF-β signaling in activated T cells (44). Th17 cells highly express the deubiquitinase ubiquitin-specific protease (USP)4, which is essential for maintaining RORγt and Th17 cell function. USP4 interacted and deubiquitinated K48-linked polyubiquitination of RORγt, thereby promoting RORγt function and *Il17a* transcription (47). Further, it was shown that USP17 stabilizes RORγt protein expression by reducing RORγt polyubiquitination at its Lys-360 residue (49). In contrast, knockdown of endogenous USP17 in Th17 cells resulted in decreased RORγt protein levels and downregulation of Th17-related genes. Furthermore, USP17 expression was upregulated in CD4^+^ T cells from systemic lupus erythematosus patients (49). USP19 was shown to suppress Th17 cells *in vitro* and Th17-mediated pathogenesis *in vivo*. Mechanistically, USP19 removed the K63-linked ubiquitin chain from RORγt lysine 313, which is essential for recruiting the coactivator SRC3 (50). In contrast, USP1 promoted Th17-cell differentiation by attenuating Treg-cell differentiation. USP1 in CD4^+^ T cells enhanced the activity of RORγt but promoted the proteasomal degradation of Foxp3 (46). USP15 interacts with RORγt and removes ubiquitin from K446, and stimulates RORγt activity by enhancing coactivator SRC1 recruitment. Knockdown of USP15 or expression of inactive USP15 impaired Th17 differentiation, suggesting a positive role for USP15-mediated deubiquitination of RORγt in Th17 differentiation (48). USP21 was shown to interact with and stabilize AhR by removing the K48-linked polyubiquitin chains from AhR (51). USP21 inhibits the transcriptional activity of AhR in a deubiquitinating-dependent manner. USP21 deubiquitinates at the K432 residue, and ubiquitination on this site is required for the transcriptional activity of AhR. Deficiency of USP21 enhanced the differentiation of Th17 cells *in vitro* and *in vivo* (51). The USP21-deficient T cells were more colitogenic upon adoptive transfer to *Rag1* ^−/−^ mice (51). Thus, IL-17A expression is tightly regulated by the ubiquitin pathway by targeting key signaling intermediates and transcription factors (**Table 1**).

### Ubiquitination in IL-17A receptor signaling

Upon binding to the IL-17RA-IL-17RC receptor complex, IL-17A activates NF-κB to induce the expression of *Il17a* target genes (5). While IL-17RA is expressed ubiquitously, IL-17RC expression is restricted, which limits IL-17A signaling to epithelial and mesenchymal cells (5). The cytoplasmic tail of the IL-17R contains a conserved SEFIR domain (52). A SEFIR domain was also found in the adaptor protein Act1, which is implicated in the activation of NF-κB (52). Subsequent work showed that Act1 was recruited to IL-17RA in an IL-17-dependent manner. Act1 contains a tumor-necrosis factor receptor-associated factor (TRAF)-binding motif that recruits TRAF6 (53, 54). This results in K63-linked ubiquitination of TRAF6, activating the kinase TAK1 and NF-κB (53, 54). Unrestrained IL-17A signaling is prevented by K48-linked ubiquitination of Act1 by F-box E3 ubiquitin ligase β-TrCP (55). A20, a deubiquitinase, also fine-tunes IL-17A signaling (56). A20 is recruited via the CBAD to IL-17RA and removes the K63-linked ubiquitin chains on TRAF6, which tempers IL-17A signaling as a negative feedback mechanism (56). Similarly, the ubiquitin-specific protease USP25 was shown to deubiquitinate TRAF6 and prevent excessive IL-17A-induced signaling and IL-17A-dependent experimental autoimmune encephalomyelitis (EAE) (57). Thus, IL-17A-mediated inflammation is prevented by the ubiquitin pathway at multiple levels.

## Conclusion

In conclusion, the regulation of IL-17-mediated inflammation by ubiquitination represents a critical layer of control in immune signaling, balancing host defense and the prevention of excessive inflammation. Ubiquitination modulates key components of IL-17A expression and signaling by targeting key signaling intermediates and transcription factors, such as RORγt and STAT3. This post-translational modification fine-tunes the intensity and duration of IL-17-driven responses, thereby shaping the overall immune milieu. Disruptions in this regulatory network are increasingly linked to the pathogenesis of autoimmune diseases, where unchecked IL-17 signaling contributes to chronic inflammation and tissue damage.

Targeting IL-17A and IL-17 receptors using antibodies (e.g., the IL-17 inhibitor secukinumab and the IL-17R inhibitor brodalumab) has achieved remarkable success in treating psoriasis (58). However, these agents have unexpectedly low efficacy in IL-17-related diseases such as rheumatoid arthritis (RA) and multiple sclerosis (MS). This was suggested to be due to autonomous activation of IL-17R signaling and resistance to IL-17-directed therapy (59). Moreover, there is a potential risk of systemic inactivation of IL-17A activity, which provides host defense and barrier function at mucosal surfaces (60–63). As a result, treatment with IL-17A inhibitors is linked to new-onset and exacerbations of inflammatory bowel disease and colitis (8).

The advancements in the understanding of ubiquitin-mediated regulation could aid in developing strategies to inhibit selective aspects of IL-17A-mediated inflammation in a site-specific manner. Targeted modulation of ubiquitin-related enzymes within the IL-17A pathway holds tremendous promise for the treatment of autoimmune disorders. Small molecules that block or promote interactions between E3 ligases and their substrates could be developed to dampen pathological IL-17 activity without broadly compromising host defense. Moreover, the development of proteolysis-targeting chimeras (PROTACs) presents an exciting avenue for increasing the specificity of substrate degradation, thereby enabling the selective removal of pro-inflammatory signaling proteins. These strategies reflect a shift toward precision immunomodulation, where leveraging the specificity of the ubiquitin system may yield next-generation therapeutics capable of restoring immune balance in autoimmune conditions. Overall, targeting the ubiquitin machinery within the IL-17 axis holds promise for precision immunomodulation, offering opportunities to mitigate chronic inflammation while preserving protective immunity.

## References

[1] Schnell A Littman DR Kuchroo VK. T(H)17 cell heterogeneity and its role in tissue inflammation. Nat Immunol. (2023) 24:19–29. doi: 10.1038/s41590-022-01387-9, PMID: 36596896 PMC10795475

[2] Patel DD Kuchroo VK. Th17 cell pathway in human immunity: lessons from genetics and therapeutic interventions. Immunity. (2015) 43:1040–51. doi: 10.1016/j.immuni.2015.12.003, PMID: 26682981

[3] Venuprasad K Zeng M Baughan SL Massoumi R. Multifaceted role of the ubiquitin ligase Itch in immune regulation. Immunol Cell Biol. (2015) 93:452–60. doi: 10.1038/icb.2014.118, PMID: 25582340

[4] Dewson G Eichhorn PJA Komander D. Deubiquitinases in cancer. Nat Rev Cancer. (2023) 23:842–62. doi: 10.1038/s41568-023-00633-y, PMID: 37935888

[5] Gaffen SL. Structure and signalling in the IL-17 receptor family. Nat Rev Immunol. (2009) 9:556–67. doi: 10.1038/nri2586, PMID: 19575028 PMC2821718

[6] Moseley TA Haudenschild DR Rose L Reddi AH. Interleukin-17 family and IL-17 receptors. Cytokine Growth Factor Rev. (2003) 14:155–74. doi: 10.1016/S1359-6101(03)00002-9, PMID: 12651226

[7] Tong Z Yang XO Yan H Liu W Niu X Shi Y. A protective role by interleukin-17F in colon tumorigenesis. PloS One. (2012) 7:e34959. doi: 10.1371/journal.pone.0034959, PMID: 22509371 PMC3324558

[8] Kumar R Theiss AL Venuprasad K. RORgammat protein modifications and IL-17-mediated inflammation. Trends Immunol. (2021) 42:1037–50. doi: 10.1016/j.it.2021.09.005, PMID: 34635393 PMC8556362

[9] Bettelli E Carrier Y Gao W Korn T Strom TB Oukka M. Reciprocal developmental pathways for the generation of pathogenic effector TH17 and regulatory T cells. Nature. (2006) 441:235–8. doi: 10.1038/nature04753, PMID: 16648838

[10] Capone A Volpe E. Transcriptional regulators of T helper 17 cell differentiation in health and autoimmune diseases. Front Immunol. (2020) 11:348. doi: 10.3389/fimmu.2020.00348, PMID: 32226427 PMC7080699

[11] Mills KHG. IL-17 and IL-17-producing cells in protection versus pathology. Nat Rev Immunol. (2023) 23:38–54. doi: 10.1038/s41577-022-00746-9, PMID: 35790881 PMC9255545

[12] McGeachy MJ Cua DJ Gaffen SL. The IL-17 family of cytokines in health and disease. Immunity. (2019) 50:892–906. doi: 10.1016/j.immuni.2019.03.021, PMID: 30995505 PMC6474359

[13] Mbow M Larkin BM Meurs L Wammes LJ de Jong SE Labuda LA. T-helper 17 cells are associated with pathology in human schistosomiasis. J Infect Dis. (2013) 207:186–95. doi: 10.1093/infdis/jis654, PMID: 23087431 PMC3571236

[14] Malard F Gaugler B Lamarthee B Mohty M. Translational opportunities for targeting the Th17 axis in acute graft-vs.-host disease. Mucosal Immunol. (2016) 9:299–308. doi: 10.1038/mi.2015.143, PMID: 26813345

[15] Zhang X Li B Lan T Chiari C Ye X Wang K. The role of interleukin-17 in inflammation-related cancers. Front Immunol. (2024) 15:1479505. doi: 10.3389/fimmu.2024.1479505, PMID: 39906741 PMC11790576

[16] Ivanov II McKenzie BS Zhou L Tadokoro CE Lepelley A Lafaille JJ. The orphan nuclear receptor RORgammat directs the differentiation program of proinflammatory IL-17+ T helper cells. Cell. (2006) 126:1121–33. doi: 10.1016/j.cell.2006.07.035, PMID: 16990136

[17] Soroosh P Wu J Xue X Song J Sutton SW Sablad M. Oxysterols are agonist ligands of RORgammat and drive Th17 cell differentiation. Proc Natl Acad Sci U.S.A. (2014) 111:12163–8. doi: 10.1073/pnas.1322807111, PMID: 25092323 PMC4143045

[18] Santori FR Huang P van de Pavert SA Douglass EF Jr. Leaver DJ Haubrich BA. Identification of natural RORgamma ligands that regulate the development of lymphoid cells. Cell Metab. (2015) 21:286–98. doi: 10.1016/j.cmet.2015.01.004, PMID: 25651181 PMC4317570

[19] Hang S Paik D Yao L Kim E Trinath J Lu J. Bile acid metabolites control T(H)17 and T(reg) cell differentiation. Nature. (2019) 576:143–8. doi: 10.1038/s41586-019-1785-z, PMID: 31776512 PMC6949019

[20] Singh AK Kumar R Yin J Brooks Ii JF Kathania M Mukherjee S. RORgammat-Raftlin1 complex regulates the pathogenicity of Th17 cells and colonic inflammation. Nat Commun. (2023) 14:4972. doi: 10.1038/s41467-023-40622-1, PMID: 37591835 PMC10435467

[21] Liu X Wang L Zhao K Thompson PR Hwang Y Marmorstein R. The structural basis of protein acetylation by the p300/CBP transcriptional coactivator. Nature. (2008) 451:846–50. doi: 10.1038/nature06546, PMID: 18273021

[22] Wang X Zhang Y Yang XO Nurieva RI Chang SH Ojeda SS. Transcription of Il17 and Il17f is controlled by conserved noncoding sequence 2. Immunity. (2012) 36:23–31. doi: 10.1016/j.immuni.2011.10.019, PMID: 22244845 PMC3270375

[23] Liu HP Cao AT Feng T Li Q Zhang W Yao S. TGF-beta converts Th1 cells into Th17 cells through stimulation of Runx1 expression. Eur J Immunol. (2015) 45:1010–8. doi: 10.1002/eji.201444726, PMID: 25605286 PMC4441226

[24] Zhang F Meng G Strober W. Interactions among the transcription factors Runx1, RORgammat and Foxp3 regulate the differentiation of interleukin 17-producing T cells. Nat Immunol. (2008) 9:1297–306. doi: 10.1038/ni.1663, PMID: 18849990 PMC4778724

[25] Durant L Watford WT Ramos HL Laurence A Vahedi G Wei L. Diverse targets of the transcription factor STAT3 contribute to T cell pathogenicity and homeostasis. Immunity. (2010) 32:605–15. doi: 10.1016/j.immuni.2010.05.003, PMID: 20493732 PMC3148263

[26] Brustle A Heink S Huber M Rosenplanter C Stadelmann C Yu P. The development of inflammatory T(H)-17 cells requires interferon-regulatory factor 4. Nat Immunol. (2007) 8:958–66. doi: 10.1038/ni1500, PMID: 17676043

[27] Hasan Z Koizumi SI Sasaki D Yamada H Arakaki N Fujihara Y. JunB is essential for IL-23-dependent pathogenicity of Th17 cells. Nat Commun. (2017) 8:15628. doi: 10.1038/ncomms15628, PMID: 28555647 PMC5460000

[28] Lebson L Gocke A Rosenzweig J Alder J Civin C Calabresi PA. Cutting edge: The transcription factor Kruppel-like factor 4 regulates the differentiation of Th17 cells independently of RORgammat. J Immunol. (2010) 185:7161–4. doi: 10.4049/jimmunol.1002750, PMID: 21076063 PMC3071015

[29] Dang EV Barbi J Yang HY Jinasena D Yu H Zheng Y. Control of T(H)17/T(reg) balance by hypoxia-inducible factor 1. Cell. (2011) 146:772–84. doi: 10.1016/j.cell.2011.07.033, PMID: 21871655 PMC3387678

[30] Venuprasad K Yang C Shao Y Demydenko D Harada Y Jeon MS. Immune regulation by ubiquitin conjugation. Adv Exp Med Biol. (2006) 584:207–17. doi: 10.1007/0-387-34132-3_15, PMID: 16802609

[31] Qu Z Fu J Ma H Zhou J Jin M Mapara MY. PDLIM2 restricts Th1 and Th17 differentiation and prevents autoimmune disease. Cell Biosci. (2012) 2:23. doi: 10.1186/2045-3701-2-23, PMID: 22731402 PMC3543335

[32] Tanaka T Yamamoto Y Muromoto R Ikeda O Sekine Y Grusby MJ. PDLIM2 inhibits T helper 17 cell development and granulomatous inflammation through degradation of STAT3. Sci Signal. (2011) 4:ra85. doi: 10.1126/scisignal.2001637, PMID: 22155789

[33] Cho JJ Xu Z Parthasarathy U Drashansky TT Helm EY Zuniga AN. Hectd3 promotes pathogenic Th17 lineage through Stat3 activation and Malt1 signaling in neuroinflammation. Nat Commun. (2019) 10:701. doi: 10.1038/s41467-019-08605-3, PMID: 30741923 PMC6370850

[34] Kathania M Khare P Zeng M Cantarel B Zhang H Ueno H. Itch inhibits IL-17-mediated colon inflammation and tumorigenesis by ROR-gammat ubiquitination. Nat Immunol. (2016) 17:997–1004. doi: 10.1038/ni.3488, PMID: 27322655

[35] Sun J Jia H Bao X Wu Y Zhu T Li R. Tumor exosome promotes Th17 cell differentiation by transmitting the lncRNA CRNDE-h in colorectal cancer. Cell Death Dis. (2021) 12:123. doi: 10.1038/s41419-020-03376-y, PMID: 33495437 PMC7835218

[36] Zeng Q Guo H Tang N Renavikar PS Karandikar NJ Lovett-Racke AE. K27-linked RORgammat ubiquitination by Nedd4 potentiates Th17-mediated autoimmunity. J BioMed Sci. (2025) 32:26. doi: 10.1186/s12929-025-01120-2, PMID: 39972304 PMC11841259

[37] Layman AAK Sprout SL Phillips D Oliver PM. Ndfip1 restricts Th17 cell potency by limiting lineage stability and proinflammatory cytokine production. Sci Rep. (2017) 7:39649. doi: 10.1038/srep39649, PMID: 28051111 PMC5209687

[38] Ramon HE Beal AM Liu Y Worthen GS Oliver PM. The E3 ubiquitin ligase adaptor Ndfip1 regulates Th17 differentiation by limiting the production of proinflammatory cytokines. J Immunol. (2012) 188:4023–31. doi: 10.4049/jimmunol.1102779, PMID: 22403444 PMC3713491

[39] Oliver PM Cao X Worthen GS Shi P Briones N MacLeod M. Ndfip1 protein promotes the function of itch ubiquitin ligase to prevent T cell activation and T helper 2 cell-mediated inflammation. Immunity. (2006) 25:929–40. doi: 10.1016/j.immuni.2006.10.012, PMID: 17137798 PMC2955961

[40] Kathania M Kumar R Lenou ET Basrur V Theiss AL Chernoff J. Pak2-mediated phosphorylation promotes RORgammat ubiquitination and inhibits colonic inflammation. Cell Rep. (2022) 40:111345. doi: 10.1016/j.celrep.2022.111345, PMID: 36103814 PMC9510046

[41] Wang X Yang J Han L Zhao K Wu Q Bao L. TRAF5-mediated lys-63-linked polyubiquitination plays an essential role in positive regulation of RORgammat in promoting IL-17A expression. J Biol Chem. (2015) 290:29086–94. doi: 10.1074/jbc.M115.664573, PMID: 26453305 PMC4661420

[42] Matsui-Hasumi A Sato Y Uto-Konomi A Yamashita S Uehori J Yoshimura A. E3 ubiquitin ligases SIAH1/2 regulate hypoxia-inducible factor-1 (HIF-1)-mediated Th17 cell differentiation. Int Immunol. (2017) 29:133–43. doi: 10.1093/intimm/dxx014, PMID: 28338984

[43] Chitrakar A Budda SA Henderson JG Axtell RC Zenewicz LA. E3 ubiquitin ligase von hippel-lindau protein promotes th17 differentiation. J Immunol. (2020) 205:1009–23. doi: 10.4049/jimmunol.2000243, PMID: 32690659 PMC8167928

[44] Rutz S Kayagaki N Phung QT Eidenschenk C Noubade R Wang X. Deubiquitinase DUBA is a post-translational brake on interleukin-17 production in T cells. Nature. (2015) 518:417–21. doi: 10.1038/nature13979, PMID: 25470037

[45] Zhong W Feng L Tian W Qu H Xu H Ning K. SMURF1 inhibits the Th17 and Th17.1 polarization and improves the Treg/Th17 imbalance in systemic lupus erythematosus through the ubiquitination of RORgammat. Mol Immunol. (2023) 157:186–94. doi: 10.1016/j.molimm.2023.03.024, PMID: 37054520

[46] Zhu X Wang P Zhan X Zhang Y Sheng J He S. USP1-regulated reciprocal differentiation of Th17 cells and Treg cells by deubiquitinating and stabilizing TAZ. Cell Mol Immunol. (2023) 20:252–63. doi: 10.1038/s41423-022-00969-9, PMID: 36600049 PMC9970968

[47] Yang J Xu P Han L Guo Z Wang X Chen Z. Cutting edge: Ubiquitin-specific protease 4 promotes Th17 cell function under inflammation by deubiquitinating and stabilizing RORgammat. J Immunol. (2015) 194:4094–7. doi: 10.4049/jimmunol.1401451, PMID: 25821221

[48] He Z Wang F Ma J Sen S Zhang J Gwack Y. Ubiquitination of RORgammat at lysine 446 limits th17 differentiation by controlling coactivator recruitment. J Immunol. (2016) 197:1148–58. doi: 10.4049/jimmunol.1600548, PMID: 27430721 PMC4976012

[49] Han L Yang J Wang X Wu Q Yin S Li Z. The E3 deubiquitinase USP17 is a positive regulator of retinoic acid-related orphan nuclear receptor gammat (RORgammat) in Th17 cells. J Biol Chem. (2014) 289:25546–55. doi: 10.1074/jbc.M114.565291, PMID: 25070893 PMC4162160

[50] Zhang J Bouch RJ Blekhman MG He Z. USP19 suppresses th17-driven pathogenesis in autoimmunity. J Immunol. (2021) 207:23–33. doi: 10.4049/jimmunol.2100205, PMID: 34135062 PMC8674369

[51] Wang L Cheng H Wang X Zhu F Tian N Xu Z. Deubiquitination of aryl hydrocarbon receptor by USP21 negatively regulates T helper 17 cell differentiation. J Leukoc Biol. (2024) 117. doi: 10.1093/jleuko/qiae148, PMID: 38952265

[52] Zhang B Liu C Qian W Han Y Li X Deng J. Crystal structure of IL-17 receptor B SEFIR domain. J Immunol. (2013) 190:2320–6. doi: 10.4049/jimmunol.1202922, PMID: 23355738 PMC3578156

[53] Qian Y Liu C Hartupee J Altuntas CZ Gulen MF Jane-Wit D. The adaptor Act1 is required for interleukin 17-dependent signaling associated with autoimmune and inflammatory disease. Nat Immunol. (2007) 8:247–56. doi: 10.1038/ni1439, PMID: 17277779

[54] Liu C Qian W Qian Y Giltiay NV Lu Y Swaidani S. Act1, a U-box E3 ubiquitin ligase for IL-17 signaling. Sci Signal. (2009) 2:ra63. doi: 10.1126/scisignal.2000382, PMID: 19825828 PMC3182834

[55] Shi P Zhu S Lin Y Liu Y Liu Y Chen Z. Persistent stimulation with interleukin-17 desensitizes cells through SCFbeta-TrCP-mediated degradation of Act1. Sci Signal. (2011) 4:ra73. doi: 10.1126/scisignal.2001653, PMID: 22045853

[56] Garg AV Ahmed M Vallejo AN Ma A Gaffen SL. The deubiquitinase A20 mediates feedback inhibition of interleukin-17 receptor signaling. Sci Signal. (2013) 6:ra44. doi: 10.1126/scisignal.2003699, PMID: 23737552 PMC4028484

[57] Zhong B Liu X Wang X Chang SH Liu X Wang A. Negative regulation of IL-17-mediated signaling and inflammation by the ubiquitin-specific protease USP25. Nat Immunol. (2012) 13:1110–7. doi: 10.1038/ni.2427, PMID: 23042150 PMC3477275

[58] von Stebut E Boehncke WH Ghoreschi K Gori T Kaya Z Thaci D. IL-17A in psoriasis and beyond: cardiovascular and metabolic implications. Front Immunol. (2019) 10:3096. doi: 10.3389/fimmu.2019.03096, PMID: 32010143 PMC6974482

[59] Luo Q Liu Y Shi K Shen X Yang Y Liang X. An autonomous activation of interleukin-17 receptor signaling sustains inflammation and promotes disease progression. Immunity. (2023) 56:2006–2020 e6. doi: 10.1016/j.immuni.2023.06.012, PMID: 37473759

[60] Deng Z Wang S Wu C Wang C. IL-17 inhibitor-associated inflammatory bowel disease: A study based on literature and database analysis. Front Pharmacol. (2023) 14:1124628. doi: 10.3389/fphar.2023.1124628, PMID: 37033665 PMC10076642

[61] Grumme L Dombret S Knosel T Skapenko A Schulze-Koops H. Colitis induced by IL-17A-inhibitors. Clin J Gastroenterol. (2024) 17:263–70. doi: 10.1007/s12328-023-01893-9, PMID: 38060157 PMC10960887

[62] Ju J Dai Y Yang J Liu C Fan L Feng L. Crohn’s disease exacerbated by IL-17 inhibitors in patients with psoriasis: a case report. BMC Gastroenterol. (2020) 20:340. doi: 10.1186/s12876-020-01474-x, PMID: 33059618 PMC7560304

[63] Philipose J Ahmed M Idiculla PS Mulrooney SM Gumaste VV. Severe *de novo* Ulcerative Colitis following Ixekizumab Therapy. Case Rep Gastroenterol. (2018) 12:617–21. doi: 10.1159/000493922, PMID: 30483039 PMC6244035

